# Wnt/β-catenin and Hedgehog pathways are involved in the inflammatory effect of Interleukin 18 on rat chondrocytes

**DOI:** 10.18632/oncotarget.20584

**Published:** 2017-08-24

**Authors:** Jiapeng Bao, Chiyuan Ma, Jisheng Ran, Yan Xiong, Shigui Yan, Lidong Wu

**Affiliations:** ^1^ Department of Orthopedics Surgery, The 2nd Affiliated Hospital, School of Medicine, Zhejiang University, Hangzhou, 310000 China

**Keywords:** Interleukin 18, chondrocyte, osteoarthritis, Wnt/β-catenin, Hedgehog

## Abstract

To investigate the inflammatory effect of Interleukin 18 (IL-18) on rat chondrocytes and the involvement of Wnt/β-catenin and Hedgehog pathways, the mRNA and protein level of matix-degrading enzymes (MMP-2, 3, 9,13 and aggrecanses) and chondrocyte-specific proteins (Collagen II and aggrecan) were evaluated by qRT-PCR and Western blot, and key protein level of Wnt/β-catenin and Hedgehog pathways including β-catenin, GSK-3β, DKK-1, IHH, SHH, and Gli-2 were evaluated by Western blot. Dickkopf-1 (DKK-1) and Cyclopamine were used as antagonist of Wnt/β-catenin and Hedgehog pathways to perform pathway inhibition tests. In addition, location and expression of β-catenin, GSK-3β, Gli-2 and Smo were assessed by Immunofluorescence microscopy. The results showed up-regulation of matix-degrading enzymes (MMP-2, 3, 9,13 and aggrecanses) and down-regulation of chondrocyte-specific proteins (Collagen II and aggrecan) at both mRNA and protein level and activation of Wnt/β-catenin and Hedgehog pathways in the inflammatory reaction on rat chondrocytes caused by IL-18 treatment was observed. As conclusion, Wnt/β-catenin and Hedgehog pathways are involved in the inflammatory effect of IL-18 *in vitro*.

## INTRODUCTION

Osteoarthritis (OA) is one of the most common world-wide chronic diseases [1]. A number of factors are believed to be related to the development of this potentially irreversible disease including aging, obesity, trauma, genetic disorders, systemic diseases, and other problems [2]. The development of osteoarthritis is believed to be multifactorial or resulted from a series of factors. Cartilage in joints aids in smooth mobility of joints [3]. Cartilage is consisting of chondrocytes and extracellular matrix secreted by chondrocytes which is rich in collagen and proteoglycan. Previous studies had demonstrated that the participation of immune system is one of the key elements in the development and progression of OA [4]. The production and secretion of pro-inflammatory cytokines such as tumor necrosis factor-α (TNF-α) and Interleukin-1ß (IL-1ß) could result in the release of matrix metalloproteinases that assist in cartilage destruction [5].

Interleukin 18 is one member of the IL-1 cytokine family, comprising 157 amino acid residues [6, 7]. IL-18 could be produced by chondrocytes, osteoblasts, FLS, and macrophages in joints [8, 9]. It is reported that IL-18 could increase release of MMP-1, MMP-3, and MMP13 from chondrocytes [10]. Another vitro study demonstrated that IL-18 may contribute to apoptosis in human articular chondrocytes [11]. Vivo study also indicates that IL-18 promotes joint inflammation [12, 13]. However, mechanism of how IL-18 works remains nuclear. It is reported in previous study that IL-18 induces inflammation through activation of the nuclear factor-kB (NF-kB), p38 mitogen activated protein kinase (MAPK) and Erk1/2 pathways [10], other pathways related to OA are poorly studied. However, it is well known that Wnt/β-catenin and Hedgehog pathways are another two important pathways related to OA [14–18]. DKK-1 and Cyclopamine work as potent antagonist of Wnt/β-catenin and Hedgehog signaling [19, 20]. Therefore, in the present study, Wnt/β-catenin and Hedgehog pathways are investigated in inflammation reaction of chondrocytes caused by IL-18 *in vitro*, and our results will help understanding of the effect of IL-18 in OA.

## RESULTS

Up regulation of MMP-2, 3, 9, 13 and aggrecanses (adamts 4/5), down regulation of Collagen II and aggrecan caused by IL-18 stimulation.

**Figure 1:**
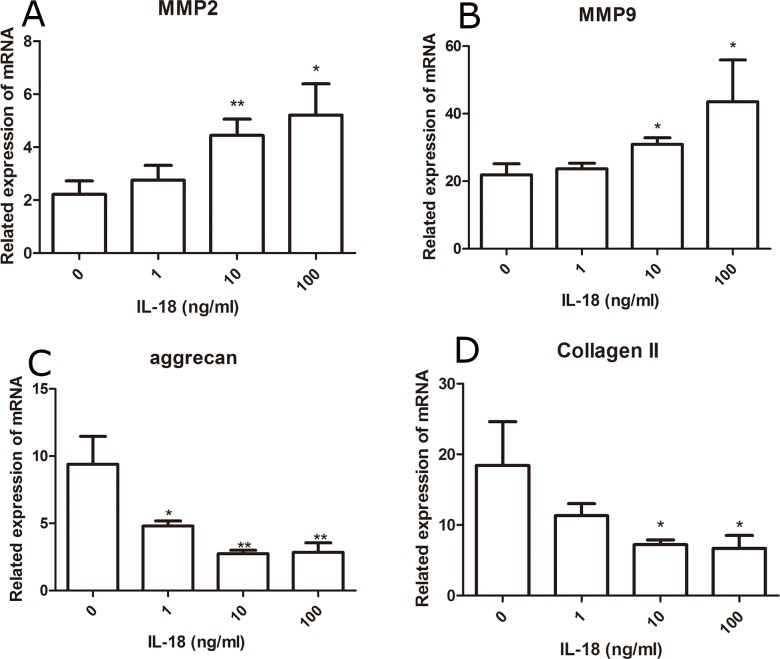
Up-regulation of matix-degrading enzyme genes and down-regulation of chondrocyte-specific genes at mRNA level caused by IL-18 were observed in SD rat chondrocytes The chondrocytes were treated with different concentrations of IL-18 for 24 hours. The expression of chondrocyte-specific genes and matix-degrading enzyme genes, MMP-2 **(A)**, MMP-9 **(B)**, aggrecan **(C)**, and Collagen II **(D)** were evaluated by Real-Time PCR. Significance was calculated by a one-way ANOVA with a post hoc Tukey's multiple comparisons test. ^*^p<0.05, ^**^p<0.01, ^***^p<0.001 versus 0ng/ml IL-18 treated group.

**Figure 2:**
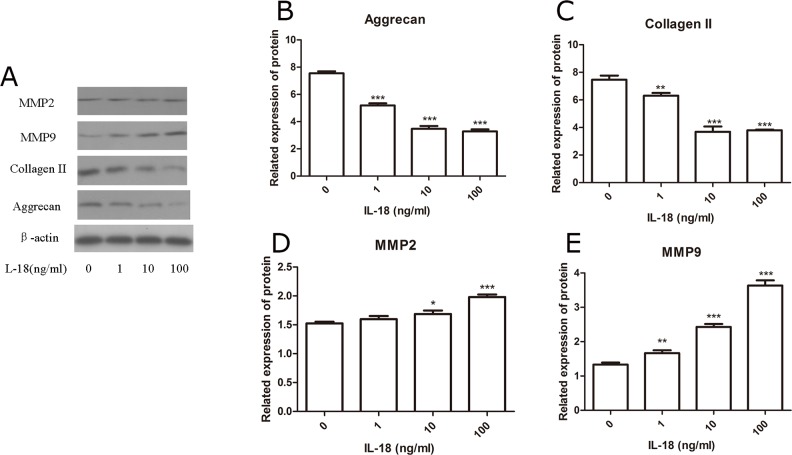
Up-regulation of matix-degrading enzymes and down-regulation of chondrocyte-specific proteins caused by IL-18 were observed in SD rat chondrocytes The chondrocytes were treated with different concentrations of IL-18 for 24 hours. The expression of chondrocyte-specific proteins and matix-degrading enzymes, MMP-2 **(D)**, MMP-9 **(E)**, aggrecan **(B)**, and Collagen II **(C)** were evaluated by Western blots **(A)**, β-actin worked as internal control here. Significance was calculated by a one-way ANOVA with a post hoc Tukey's multiple comparisons test. ^*^p<0.05, ^**^p<0.01, ^***^p<0.001 versus 0ng/ml IL-18 treated group.

We performed qRT-PCR to detect the expression levels of MMP-2, 3, 9, 13, aggrecanses (adamts 4/5) Collagen II and aggrecan in chondrocytes. Chondrocytes were treated with IL-18 in different concentration (0ng/ml as control, 1ng/ml, 10ng/ml, 100ng/ml) for 24 hours. RT-PCR showed significant up-regulation of mRNA levels of MMP-2, 3, 9, 13 adamts 4/5, down-regulation of mRNA levels of Collagen II and aggrecan, which is IL-18 dose-dependent (Figure 1, Supplementary Figure 1A). Then, we assessed the protein level of MMP-2, 3, 9, 13 adamts 4/5, Collagen II and aggrecan by Western blot analysis (Figure 2, Supplementary Figure 1B), which comes to the similar results of qRT-PCR. All these results suggested the inflammatory role of IL-18 on cartilage matrix by up-regulation of matrix-degrading enzymes and by down-regulation of matrix components secreted chondrocytes *in vitro*, which is similar with the results of previous studies [10, 11].

**Figure 3:**
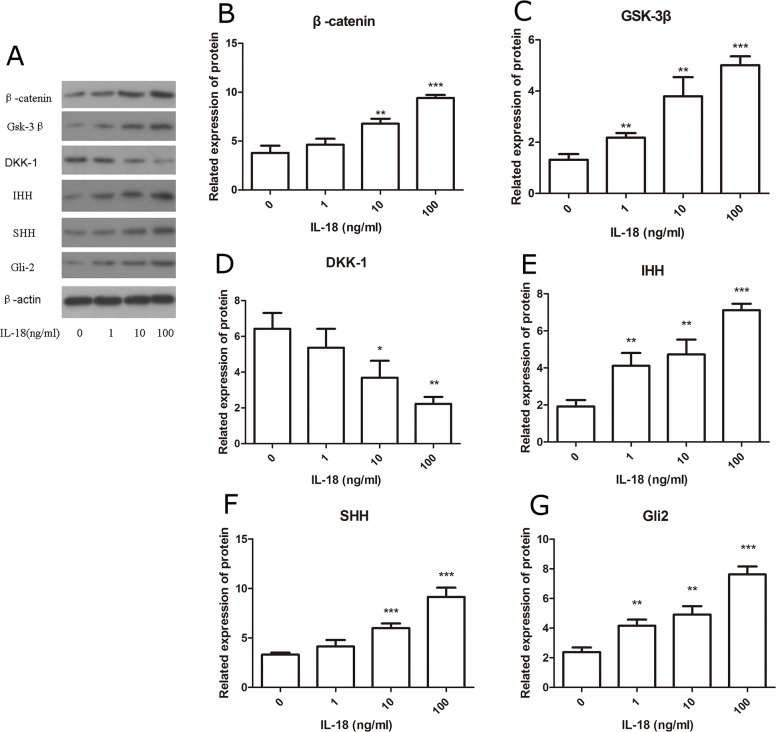
IL-18 stimulation increased the protein level of Wnt pathway related proteins and Hedgehog related proteins The chondrocytes were treated with different concentrations of IL-18 for 24 hours. The level of β-catenin **(B)**, GSK-3β **(C)**, DKK-1 **(D)**, IHH **(E)**, SHH **(F)**, Gli-2 **(G)**, and β-actin in total extract was investigated by Western blot **(A)**. Significance was calculated by a one-way ANOVA with a post hoc Tukey's multiple comparisons test. ^*^p<0.05, ^**^p<0.01, ^***^p<0.001 versus 0ng/ml IL-18 treated group.

**Figure 4:**
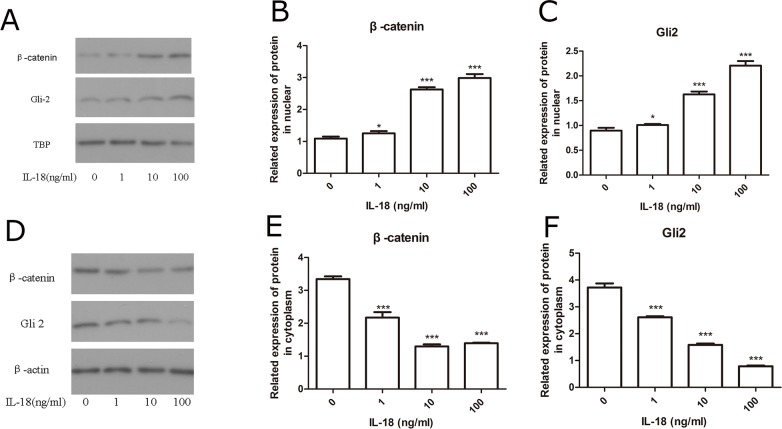
IL-18 induced nuclear translocation of β-catenin and Gli-2 The chondrocytes were treated with different concentrations of IL-18 for 24 hours. The level of β-catenin and Gli2 in nucleus **(A-C)** and cytosol **(D-F)** was evaluated dividedly by Western blot. Significance was calculated by a one-way ANOVA with a post hoc Tukey's multiple comparisons test. ^*^p<0.05, ^**^p<0.01, ^***^p<0.001 versus 0ng/ml IL-18 treated group.

IL-18 stimulation increases the levels of β-catenin, GSK-3β, DKK-1, IHH, SHH, and Gli-2. β-catenin, GSK-3β, DKK-1 are several key proteins in Wnt/β-catenin pathway, and IHH, SHH, and Gli-2 play key roles in Hedgehog pathway. The amounts of the proteins in cell nucleus and whole cell after 24h IL-18 stimulation were investigated dividedly. The results of Western blot showed the significant increased protein level of β-catenin, GSK-3β, DKK-1, IHH, SHH, and Gli-2. β-catenin, GSK-3β and significant decreased protein level of DKK-1 in chondrocytes treated with IL-18 at concentrations (10ng/ml, 100ng/ml) for 24 hours when compared with control, while low concentration of IL-18 (1ng/ml) treatment couldn't provide significant change (Figure 3). Furthermore, IL-18 induced nuclear translocation of β-catenin and Gli-2, which is also IL-18 dose-dependent (Figure 4). Those results suggested that IL-18 stimulation *in vitro* had sitimulation on the Wnt/β-catenin and Hedgehog pathways and we regarded 100ng/ml as the concentration of IL-18 stimulation in the following inhibition experiments.

**Figure 5:**
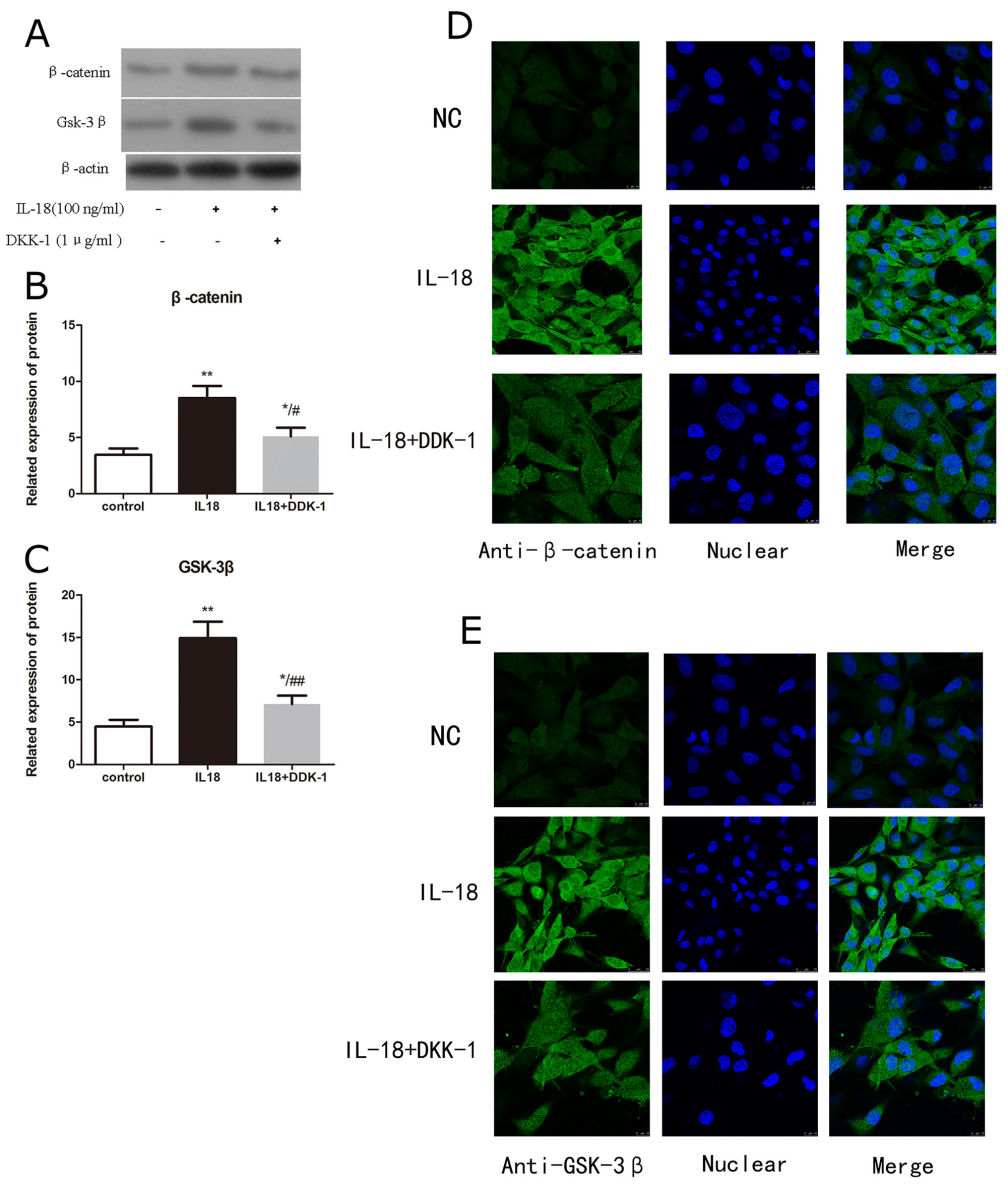
DKK-1 worked as a potent antagonist of Wnt signaling and the block effect of DKK-1 was analyzed here Chondrocytes of IL-18+DKK-1 group were pre-treated with DKK-1 (1μg/ml) for 1 hour, followed with 24h IL-18 stimulation (100ng/ml). Chondrocytes of IL-18 group were treated with 100ng/ml IL-18 for 24 hours. The level of β-catenin **(B)** and GSK-3β **(C)** in total extract was investigated by Western blot **(A)**. Significance was calculated by a one-way ANOVA with a post hoc Tukey's multiple comparisons test. ^*^p<0.05, ^**^p<0.01, ^***^p<0.001 versus control group. ^#^p<0.05, ^##^p<0.01, ^###^p<0.001 versus 100ng/ml IL-18 treated group. The location and concentration of β-catenin **(D)** and GSK-3β **(E)** was visualized using immunofluorescence analysis.

**Figure 6:**
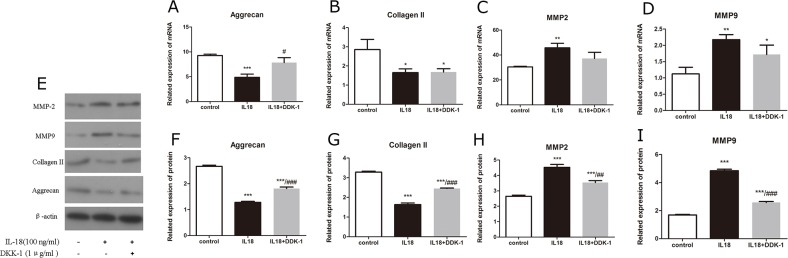
DKK-1 down-regulates IL-18-induced expression of matrix-degrading encymes Chondrocytes of IL-18+DKK-1 group were pre-treated with DKK-1 (1μg/ml) for 1 hour, followed with 24h IL-18 stimulation (100ng/ml). Chondrocytes of IL-18 group were treated with 100ng/ml IL-18 for 24 hours. The expression of chondrocyte-specific proteins and matix-degrading enzymes, MMP-2, MMP-9, aggrecan, and Collagen II were evaluated by Real-Time PCR at mRNA level **(A-D)** and by Western blot at protein level **(E-I)**. Significance was calculated by a one-way ANOVA with a post hoc Tukey's multiple comparisons test. ^*^p<0.05, ^**^p<0.01, ^***^p<0.001 versus control group. ^#^p<0.05, ^##^p<0.01, ^###^p<0.001 versus 100ng/ml IL-18 treated group

The up-regulation of MMPs, aggrecanses and down-regulation of Collagen II and aggrecan caused by IL-18 stimulation were partly inhibited by the treatment of DKK-1 (1μg/ml). We performed a series of pathway inhibition tests to proof the activiation of Wnt/β-catenin pathway. It's reported that DKK-1 works as a potent antagonist of Wnt signaling [19] and the block effect of DKK-1 was analyzed here. Immunofluorescence microscopy showed the activation of Wnt/β-catenin pathway resulted from IL-18 stimulation was inhibited by DKK-1 treatment (Figure 5). We used qRT-PCR and Western blot analysis to assess the mRNA level and protein level of MMP-2, 3, 9, 13, adamts 4, 5, Collagen II and aggrecan changes caused by 24h IL-18 stimulation (100ng/ml) after 1 hour treatment of DKK-1 (1μg/ml) (Figure 6, Supplementary Figure 2). Results demonstrated that DKK-1 could decrease the up-regulation of MMPs, aggrecanses and down-regulation of Collagen II and aggrecan caused by IL-18 stimulation. These results indicate that IL-18 induces inflammation in chondrocytes *in vitro* by activating the Wnt/β-catenin pathway.

**Figure 7:**
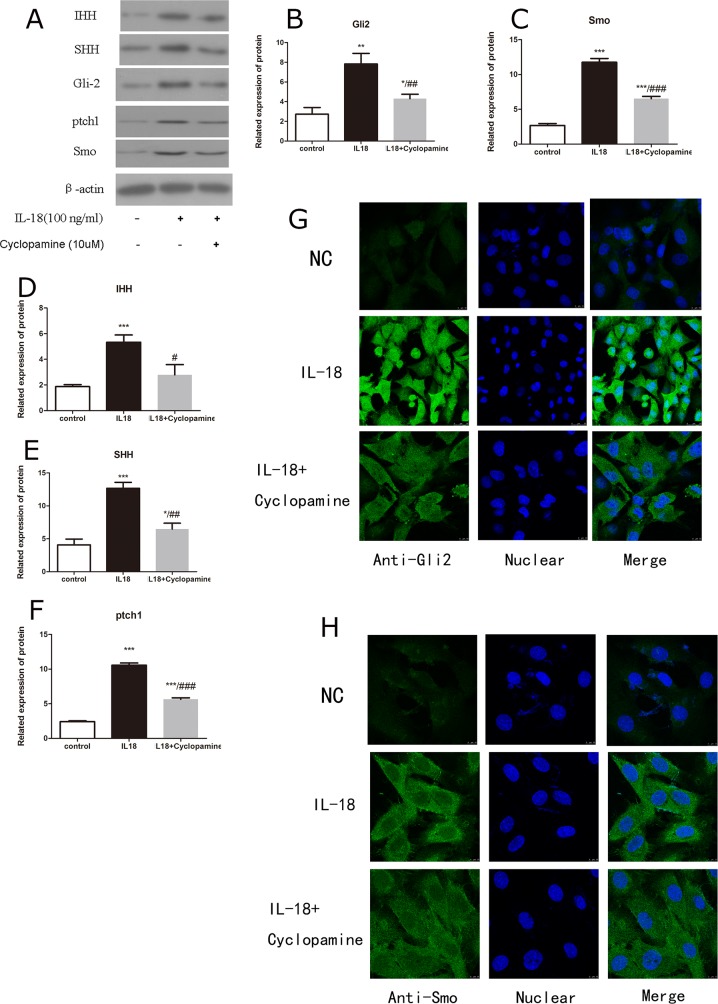
Cyclopamine worked as a potent antagonist of Hedgehog pathway Chondrocytes of IL-18+ Cyclopamine group were pre-treated with Cyclopamine (10μM) for 1 hour, followed with 24h IL-18 stimulation (100ng/ml). Chondrocytes of IL-18 group were treated with 100ng/ml IL-18 for 24 hours. The level of IHH **(D)**, SHH **(E)**, ptch1 **(F)**, Gli2 **(B)** and Smo **(C)** in total exact was investigated by Western blot **(A)**. Significance was calculated by a one-way ANOVA with a post hoc Tukey's multiple comparisons test. ^*^p<0.05, ^**^p<0.01, ^***^p<0.001 versus control group. ^#^p<0.05, ^##^p<0.01, ^###^p<0.001 versus 100ng/ml IL-18 treated group. The location and concentration of Gli2 **(G)** and Smo **(H)** was visualized using immunofluorescence analysis.

**Figure 8:**
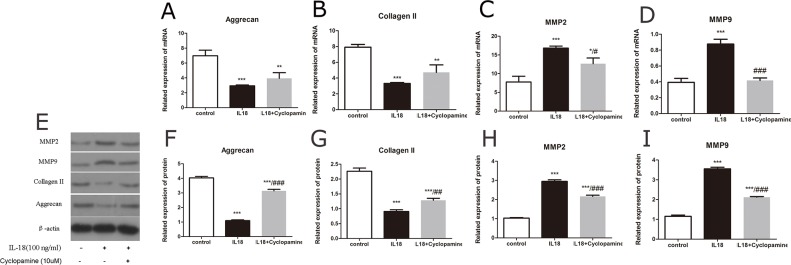
Cyclopamine down-regulates IL-18-induced expression of matrix-degrading encymes Chondrocytes of IL-18+ Cyclopamine group were pre-treated with Cyclopamine (10μM) for 1 hour, followed with 24h IL-18 stimulation (100ng/ml). Chondrocytes of IL-18 group were treated with 100ng/ml IL-18 for 24 hours. The expression of chondrocyte-specific proteins and matix-degrading enzymes, MMP-2, MMP-9, aggrecan, and Collagen II were evaluated by Real-Time PCR at mRNA level **(A-D)** and by Western blot at protein level (E-I). Significance was calculated by a one-way ANOVA with a post hoc Tukey's multiple comparisons test. ^*^p<0.05, ^**^p<0.01, ^***^p<0.001 versus control group. ^#^p<0.05, ^##^p<0.01, ^###^p<0.001 versus 100ng/ml IL-18 treated group

The up-regulation of MMPs, aggrecanses and down-regulation of Collagen II and aggrecan caused by IL-18 stimulation were partly inhibited by the treatment of Cyclopamine (10μM). To testfy that the Hedgehog pathway is involved in the IL-18 inflammatory effect, we investigated the blocking effect of Hedgehog. It's reported that cyclopamine is a potent antagonist of Hedgehog pathway [20]. Chondrocytes were first treated with Cyclopamine (10μM) for 1h, and then incubated with IL-18 (100ng/ml) for 24h before analysis. Immunofluorescence microscopy showed the activation of Hedgehog pathway resulted from IL-18 stimulation was inhibited by Cyclopamine treatment (Figure 7). Western blot and qRT-PCR were used to analyze the mRNA level and protein level of MMP-2, 3, 9, 13, adamts 4, 5, Collagen II and aggrecan changes caused by treatment. Results demonstrated that Cyclopamine could decrease the up-regulation of MMPs, aggrecanses and down-regulation of Collagen II and aggrecan caused by IL-18 stimulation (Figure 8, Supplementary Figure 3), which demonstrated that Hedgehog pathway is involved in the IL-18 inflammatory effect in chondrocytes.

## DISCUSSION

IL-18 is a cytokine with a series of biological actions. The up-regulated expression of IL-18 in cultured chondrocytes stimulated by IL-1β has been noted [8]. As a member of the IL-1 cytokine family, IL-18 is mainly produced by chondrocytes, osteoblasts, FLS, and macrophages in joints [8, 9], and the increased concentration of IL-18 in the synovial fluid, synovium, cartilage, and blood serum is reported to be related to the degree of severity of OA seen in radiographic images [21–23]. Several studies have confirmed the inflammatory effect of IL-18 on chondrocytes *in vitro* and *in vivo* [8, 10–12]. Furthermore, the previous study has found that the enhancement of MMP-1, MMP-3, and MMP-13 depended on the activation of the NF-kB and p38 MAPK pathways, whereas the increased production of TIMP-1 depended on the Erk1/2 pathway [10].

In general, cartilage degradation is mediated by MMP family, which is indeed well accepted [24]. High level of MMPs is regarded as one of the main expressions of OA [25, 26], as well as low level of aggrecan and type II collagen [27]. Proinflammatory cytokines induce expression of matix-degrading enzymes that degrade cartilage matrix and aggravate OA [28]. In addition, these cytokines downregulate the expression of genes involved in maintenance of cartilage matrix (like aggrecan and type II collagen), and promote hypertrophy and apoptosis of chondrocytes [29]. In the present study, up regulation of MMP-2, 3, 9, 13 and aggrecanses (adamts 4/5), down regulation of Collagen II and aggrecan caused by IL-18 stimulation were observed by qRT-PCR and Western blot, thus we confirmed the inflammatory effect of IL-18.

Besides NF-kB, PI3K, p38 MAPK and Erk1/2 pathways [10, 30, 31], the regulation of chondrocyte-specific proteins and matix-degrading enzymes is also related to some other pathways. Ma B et al reported that the expression of MMPs could be regulated by Wnt/β-catenin pathway [32]. A study by Xu HG et al demonstrated that intermittent cyclic mechanical tension loading led to Wnt/β-catenin signaling activation and loss of chondrogenic phonetype of chondrocytes [17]. Another recent study performed by Cheleschi S et al reported that hydrostatic pressure restores the expression levels of some miRNAs and downregulates some matix-degrading enzymes via the Wnt/β-catenin pathway [16]. Study performed by Lin AC et al demonstrated that Hedgehog signaling regulates normal chondrocyte and differentiation, and they found Hedgehog signaling is activated in osteoarthritis [18]. Meanwhile, our previous studies also indicated that several drugs could provide chondroprotective effects on OA by inhibiting Wnt/β-catenin and Hedgehog pathways [33, 34]. In this study, we investigated the role of Wnt/β-catenin and Hedgehog pathways in the proinflammatory effect of IL-18 on OA. Results of Western blot showed increased level of β-catenin, GSK-3β, DKK-1, IHH, SHH, Gli-2, ptch1, and Smo in whole cell caused by IL-18 stimulation, which means the inflammatory effect of IL-18 may be related to Wnt/β-catenin and Hedgehog pathways. As β-catenin and Gli-2 were transported into cell nucleus when the pathways were activated, the amounts of β-catenin and Gli-2 in cell nucleus were investigated in addition to confirm the activation of Wnt/β-catenin and Hedgehog pathways, and results of Western blot showed increased amounts of both β-catenin and Gli-2 in cell nucleus. In order to confirm the connection between the pathways and inflammatory effect of IL-18 on chondrocyte, we ran plenty of pathway inhibition tests. DKK-1 that was used as inhibitor of Wnt/β-catenin pathway downregulated MMP-2 and MMP-9 and upregulated aggrecan in the presence of IL-18 according to the results of qRT-PCR and Western blot. We also noticed that the results of qRT-PCR and Western blot of Collagen II was a little different (Figure 6). According to a study published in 2015, the Wnt signaling orchestrates a rich post-transcriptional regulatory function [35], so the different expression level of mRNA and protein of Collagen II may be related to the post-transcriptional regulatory function of Wnt signaling. On the other hand, we used Cyclopamine as blocker of Hedgehog signaling. Resutlts showed that Cyclopamine treatment decreased MMP-2 and MMP-9 and increased Collagen II and aggrecan in the presence of IL-18, and Cyclopamine also downregulated the amount of Gli-2 in cell nucleus. As the relationship and distribution of IL-18Rs and the effect of IL-18 on cell proliferation and apoptosis have been investigated in other studies, so we didn't run similar researches [8, 11]

Collectively, our results demonstrated that Wnt/β-catenin and Hedgehog pathways were involved in the inflammatory effect of IL-18 *in vitro*. These findings would help further understanding of IL-18 action.

## MATERIALS AND METHODS

### Materials

Recombinant rat IL-18 and rat DKK-1 as Wnt/β-catenin inhibitor were purchased from R&D Systems, Abingdon, UK. Dulbecco's Modified Eagle's Medium (DMEM), penicillin and streptomycin, fetal bovine serum (FBS), 0.25% trypsin were obtained from Gibco RRL, Grand Island, NY, USA. Cyclopamine as Hedgehog inhibitor and collagenase II were purchased from Sigma-Aldrich, St, Louis, MO, USA.

### Cell culture and treatment

This study was approved by the Ethics Committee of the 2^nd^ Affiliated Hospital, School of medicine, Zhejiang University, Hangzhou, China. Four-week-old Sprague Dawley rats were sacrificed, and cartilage harvested from the knee joints was digested immediately with 0.25% pancreatic enzymes for 30 min to remove other tissues and cells. Then, the cartilage fragments were digested with 0.2% collagenase II on a horizontal shaker at 37°C for 4 hours to isolate the cells in cartilage. The chondrocytes were grown in DMEM with 10% FBS, 100U/mL penicillin, and 100mg/mL streptomycin at 37°C with 5% CO2.. Cells were seeded in six-well plates for analysis. When studying the inflammatory effect of IL-18, Subconfluent cells were treated with IL-18 in various concentrations (0ng/ml as control, 1ng/ml, 10ng/ml, 100ng/ml) for 24 hours before analysis. According to the results, we chose 100ng/ml as the concentration of IL-18 in the following pathway inhibition tests. In pathway inhibition tests, subconfluent cells were pretreated with DKK-1 (1μg/ml) or Cyclopamine(10μm) for 1h then incubated with IL-18 (100ng/ml) for 24 hours before analysis.

### RNA extraction and Real-Time PCR

TRIzol reagent (Invitrogen, Carlsbad, CA, USA) was used to isolate RNA from chondrocytes accordingly. Total RNA was used to synthesize cDNA by reverse transcription (cDNA synthesis kit, Takara). Power SYBR Green PCR Master Mix (Applied Biosystems) was used for real-time PCR. The expression of MMP2, MMP9, Collagen II, and aggrecan was analyzed using primer sequences listed in Table 1. Primer sequences listed in Supplementary Table 1 were used to analyze the mRNA level of MMP3, MMP13, Adamts4 and Adamts5. For real-time PCR, 20 μL reaction mixture contained SYBR Green and each primer. PCR was set using StepOnePlus system (Applied Biosystems). The program included 1 cycle of denaturation at 95°C for 1 min and 40 cycles of denaturation at 95°C for 15 s, primer annealing, and extension at 63°C for 25 s, followed by melt curve analysis. Data were analyzed for fold difference using formula: 2^(CT GAPDH-CT targeted gene)^ ×10.

**Table 1:** Primers used for Real-Time PCR

Primer Sequences (5’–3’)			
Gene^*^	Forward	Reverse	Amplicon Size (bp)
Rat MMP2	GTACTGGACCCACGCCTACACT	CGCCAAATAAACCGATCCTTGA	124
Rat MMP9	GCAAACCCTGCGTATTTCCAT	GATAACCATCCGAGCGACCTTT	73
Rat Collagen II	GAGTGGAAGAGCGGAGACTACTG	GTCTCCATGTTGCAGAAGACTTTCA	83
Rat aggrecan	CTAGCTGCTTAGCAGGGATAACG	GATGACCCGCAGAGTCACAAAG	110
Rat GAPDH	GAAGGTCGGTGTGAACGGATTTG	CATGTAGACCATGTAGTTGAGGTCA	127

^*^ Rat means species is rat

### Western blot analysis

After treatment, whole cell extract was prepared using radioimmunoprecipitation assay (RIPA) containing protease and phosphatase inhibitors according to the manufacturer's protocol. Nuclear and cytosolic extracts were prepared using NE-PER Nuclear and Cytoplasmic Extraction Reagents (Pierce Biotechnology, Rockford, IL, USA). Equal amounts of cell extract were separated by 10% SDS-PAGE, and electro-transferred to polyvinylidene difluoride membranes. After blocked with 7% nonfat milk for 2 hours, the membranes were blotted with primary antibodies at 4°C overnight, incubated for 1 hour with secondary antibody. In this study, antibodies (Ab) against β-catenin (Abcam ab32572), glycogen synthase kinase-3 beta (GSK-3β, Abcam ab93926), DKK-1 (Abcam ab109416), india hedgehog (IHH, Abcam ab52919), sonic hedgehog (SHH, Abcam ab202342), GLI family zinc finger 2 (Gli-2, Santa cruz SC-28674), β-actin (Santa Cruz SC-47778), patch 1 (ptch1, Santa cruz SC-9016), TATA-box binding protein (TBP, Abcam ab197874), and smoothened (Smo, Santa cruz SC-13943) were used. Finally, signals were detected using West Dura Extended Duration Substrate with exposure to X-ray film.

### Immunofluorescence microscopy

Coexpression of key proteins of Wnt/β-catenin and Hedgehog pathways was carried out using fluorochrome-conjugated antibodies. Chondrocytes cultured on glass coverslips were fixed in 4% paraformaldehyde for 10 min, and permeabilized for 5 min with 0.1% v/v Triton X-100. Cells were incubated with primary antibody for 4 h, washed, and then incubated with fluorochrome-conjugated secondary Ab for 2 h in the dark. Coverslips were mounted onto glass slides using DAPI-containing mounting medium.

### Statistical analysis

All experiments were repeated three times. All the data in figures are presented as a grouped column scatter of three repeats with diplayed mean. Statistical differences were performed with SPSS 12.0 version. One-way ANOVA with a subsequent post hoc Tukey's test was used for multiple comparisons. P<0.05 is considered to be significant with statistical meaning.

## SUPPLEMENTARY MATERIALS FIGURES



## Abbreviations

IL-18: Interleukin 18
DKK-1: Dickkopf-1
FBS: fetal bovine serum
MMP: matrix metalloproteinase
Ab: antibodies
GSK-3β: glycogen synthase kinase-3 beta
IHH: india hedgehog
SHH: sonic hedgehog
Gli-2: GLI family zinc finger 2
ptch 1: patch 1
TBP: TATA-box binding protein
Smo: smoothened
NF-kB: nuclear factor-kB
MAPK: mitogen activated protein kinase
OA: Osteoarthritis
