# Supplementary material for: Wnt/β-catenin and Hedgehog pathways are involved in the inflammatory effect of Interleukin 18 on rat chondrocytes
Source: Oncotarget. 2017 Aug 24;8(66):109962–72. doi: 10.18632/oncotarget.20584 (PMC5746357; doi:10.18632/oncotarget.20584)
Supplement: Supplementary file 1 [file oncotarget-08-109962-s001.pdf]

Wnt/β-catenin and Hedgehog pathways are involved in the inflammatory effect of Interleukin 18 on rat chondrocytes

SUPPLEMENTARY MATERIALS

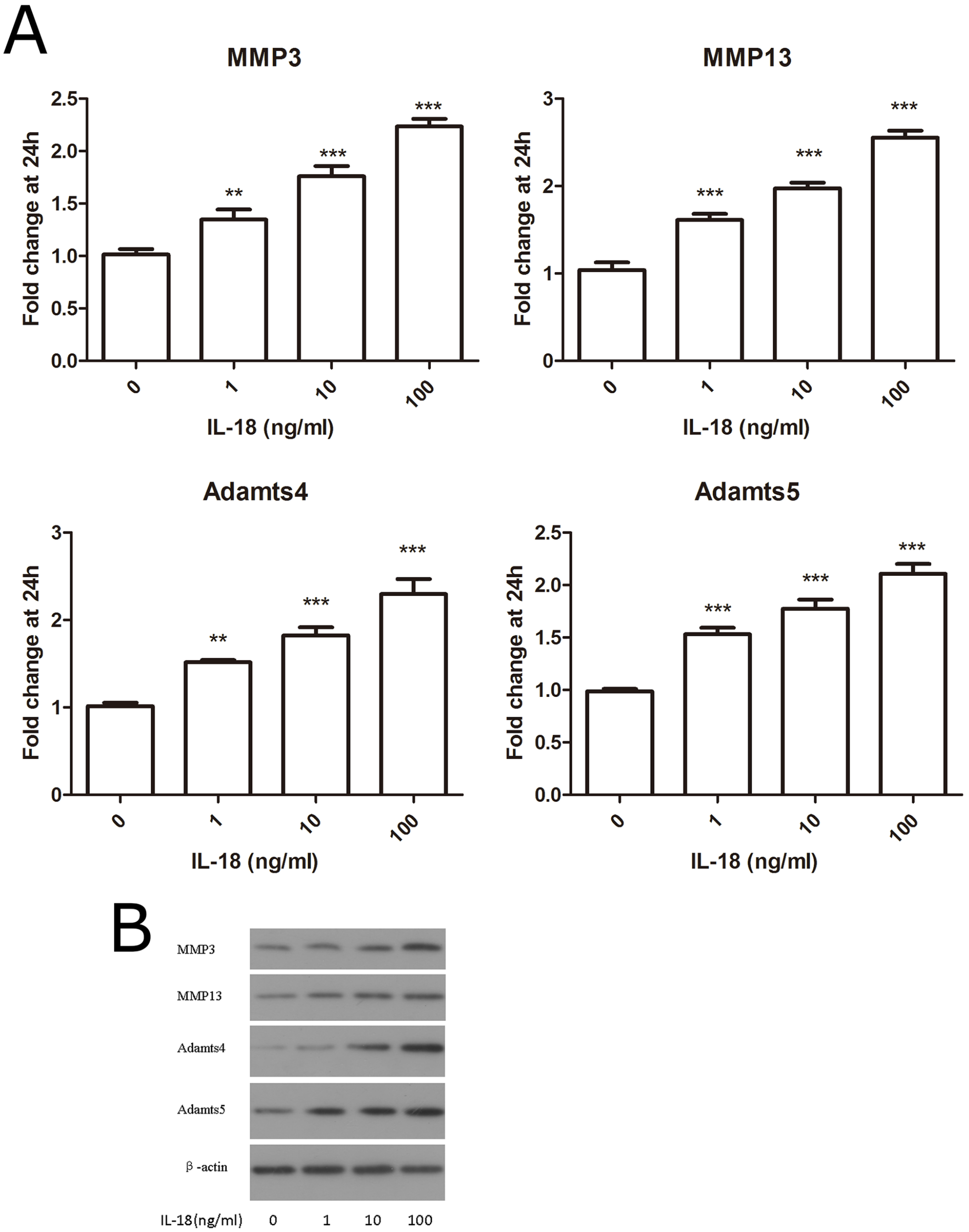

Supplementary Figure 1: Up-regulation of MMP3/13 and Adamts4/5 caused by IL-18 were observed in SD rat chondrocytes. The chondrocytes were treated with different concentrations of IL-18 for 24 hours. The expression of MMP3/13 and Adamts4/5 was evaluated by Real-Time PCR at mRNA level **(A)** and Western blots at protein level **(B)**. Significance was calculated by a one-way ANOVA with a post hoc Tukey's multiple comparisons test. $^{**}p<0.01$, $^{***}p<0.001$ versus 0ng/ml IL-18 treated group.

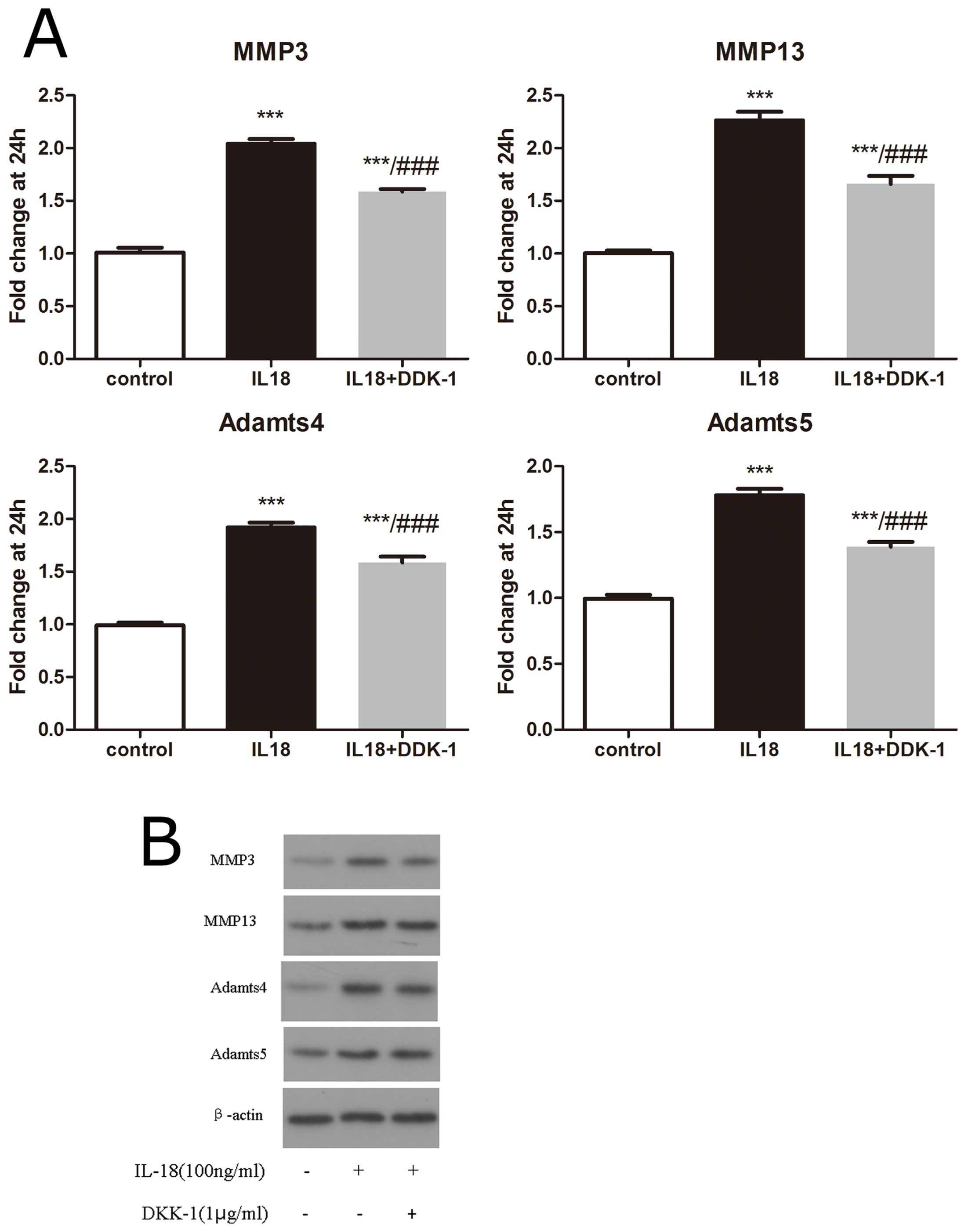

Supplementary Figure 2: DKK-1 down-regulates IL-18-induced expression of MMP3/13 and Adamts4/5. Chondrocytes of IL-18+DKK-1 group were pre-treated with DKK-1 (1μg/ml) for 1 hour, followed with 24h IL-18 stimulation (100ng/ml). Chondrocytes of IL-18 group were treated with 100ng/ml IL-18 for 24 hours. The expression of MMP3/13 and Adamts4/5 was evaluated by Real-Time PCR at mRNA level **(A)** and by Western blot at protein level **(B)**. Significance was calculated by a one-way ANOVA with a post hoc Tukey's multiple comparisons test. $^{***}p<0.001$ versus control group. $^{\#\#\#}p<0.001$ versus 100ng/ml IL-18 treated group

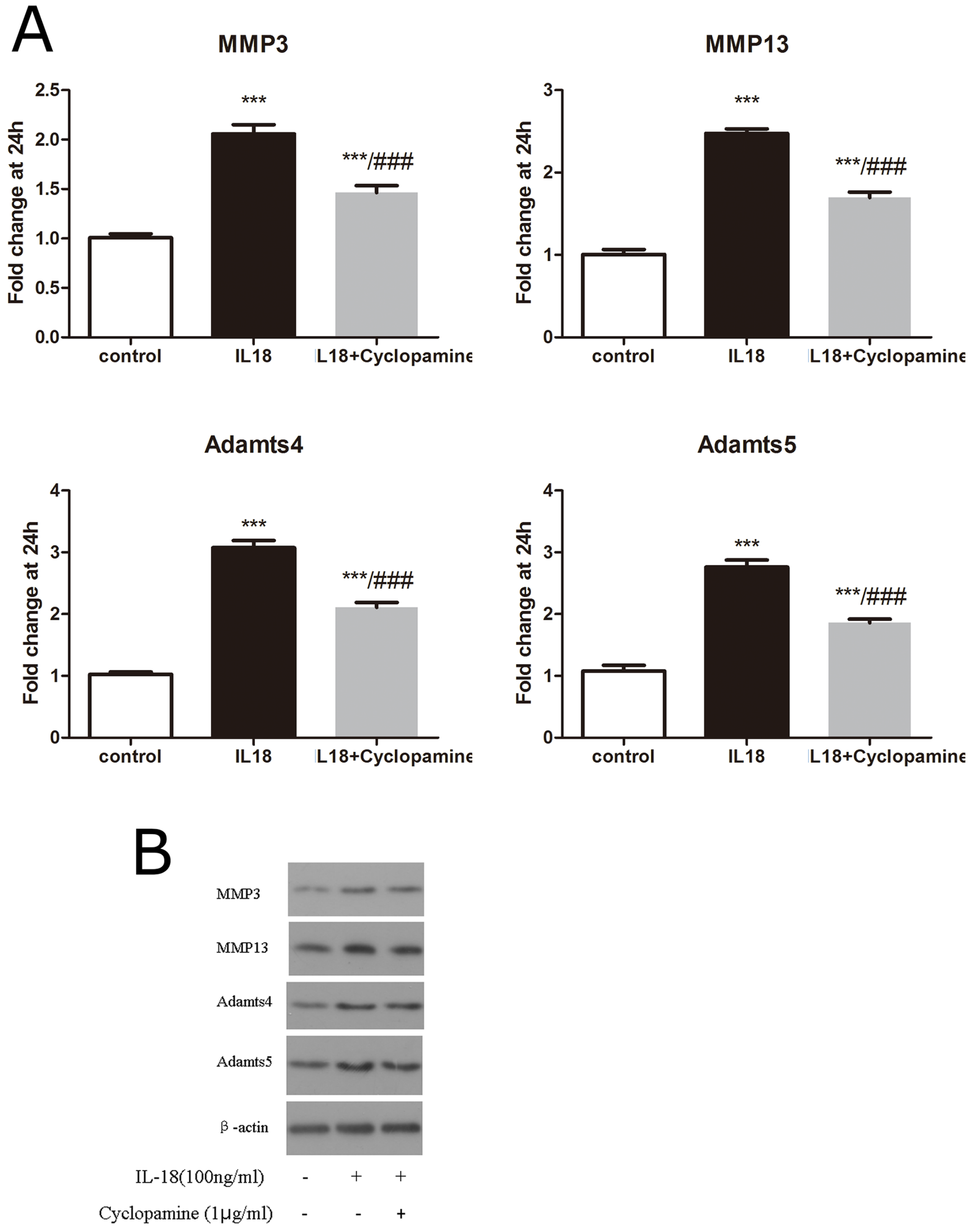

Supplementary Figure 3: Cyclopamine down-regulates IL-18-induced expression of MMP3/13 and Adamts4/5. Chondrocytes of IL-18+ Cyclopamine group were pre-treated with Cyclopamine (10μM) for 1 hour, followed with 24h IL-18 stimulation (100ng/ml). Chondrocytes of IL-18 group were treated with 100ng/ml IL-18 for 24 hours. The expression of MMP3/13 and Adamts4/5 was evaluated by Real-Time PCR at mRNA level **(A)** and by Western blot at protein level **(B)**. Significance was calculated by a one-way ANOVA with a post hoc Tukey's multiple comparisons test. ***p<0.001 versus control group. $^{\#\#\#}$p<0.001 versus 100ng/ml IL-18 treated group

Supplementary Table 1: Primers used for Real-Time PCR

	Primer Sequences (5'–3')		
Gene*	Forward	Reverse	Amplicon Size (bp)
Rat MMP3	CTGGGCTATCCGAGGTCATG	TGGACGGTTTCAGGGAGGC	124
Rat MMP13	CAACCCTGTTTACCTACCCACTTAT	CTATGTCTGCCTTAGCTCCTGTC	73
Rat Adamts4	GCCAGCAACCGAGGTCCCATA	CCACCACCAGTGTCTCCACGAAT	83
Rat Adamts5	GCCACGACCCTCAAGAACTTTT	CAGGATGGCTGCATCGTAGT	110
Rat GAPDH	GAAGGTCGGTGTGAACGGATTTG	CATGTAGACCATGTAGTTGAGGTCA	127

* Rat means species is rat
